# Developing a One-Stop Platform Transportation Planning Service to Help Older Adults Move Around in Their Community Where, When, and How They Wish: Protocol for a Living Lab Study

**DOI:** 10.2196/33894

**Published:** 2022-06-09

**Authors:** Veronique Provencher, Dany Baillargeon, Bessam Abdulrazak, Patrick Boissy, Mélanie Levasseur, Nathalie Delli-Colli, Hélène Pigot, Mélisa Audet, Sara Bahrampoor Givi, Catherine Girard

**Affiliations:** 1 School of Rehabilitation Université de Sherbrooke Sherbrooke, QC Canada; 2 Research Center on Aging Centre intégré universitaire de santé et de services sociaux de l'Estrie - Centre hospitalier universitaire de Sherbrooke Sherbrooke, QC Canada; 3 Department of Communication Université de Sherbrooke Sherbrooke, QC Canada; 4 Department of Computer Science Université de Sherbrooke Sherbrooke, QC Canada; 5 Department of Surgery-Orthopedics Université de Sherbrooke Sherbrooke, QC Canada; 6 School of Social Work Université de Sherbrooke Sherbrooke, QC Canada; 7 Université de Sherbrooke Sherbrooke, QC Canada

**Keywords:** aged, mobility limitation, transportation, community-based, participatory research, information system, mobility, older adults

## Abstract

**Background:**

Multiple mobility-related challenges frequently appear with aging. As a result, many older adults have difficulty getting around, to go, for example, to doctors’ appointments or leisure activities. Although various means of transportation are currently available, older adults do not necessarily use them, partly because they do not know which ones are adapted to their needs and preferences. To foster older adults’ autonomy and freedom in their decision-making about transportation, it is crucial to help them make informed decisions about the means that suit them best.

**Objective:**

Our aim is to develop Mobilainés, a one-stop platform transportation planning service combining different transport modes and services to help older adults move around in their community where, when, and how they wish. More specifically, we aim to (1) define older adults’ mobility needs and preferences in order to conceptualize a one-stop platform; (2) cocreate a prototype of the one-stop platform; and (3) test the prototype with users in a real-life context.

**Methods:**

This ongoing study uses a “Living Lab” co-design approach. This approach differs from traditional research on aging by facilitating intersectoral knowledge sharing and innovative solutions by and with older adults themselves. A steering committee of 8 stakeholders from the public, scientific, and private sectors, as well as older citizens, will meet quarterly throughout the study. The design comprises three phases, each with several iterative subphases. Phase 1 is exploration: through co-design workshops and literature reviews, members of the intersectoral committee will define older adults’ mobility needs and preferences to support the conceptualization of the one-stop platform. Phase 2 is experimentation: 4 personas will be produced that reflect the different needs and preferences of typical older adult end users of the platform; for development of a prototype, scenarios and mockups (static designs of the web application) will be created through co-design sessions with older adults (N=12) embodying these personas. Phase 3 is evaluation: we will test the usability of the prototype and document changes in mobility, such as the ability to move around satisfactorily and to participate in meaningful activities, by and with older adults (N=30) who use the prototype. The steering committee will identify ways to support the adoption, implementation, and scaling up of Mobilainés to ensure its sustainability. Qualitative and quantitative data will be triangulated according to each subphase objective.

**Results:**

The first phase began in September 2019. The study is scheduled for completion by mid-2023.

**Conclusions:**

This innovative transportation planning service will merge existing transportation options in one place. By meeting a wide variety of older adults’ needs and preferences, Mobilainés will help them feel comfortable and safe when moving around, which should increase their participation in meaningful activities and reduce the risk of social isolation.

**International Registered Report Identifier (IRRID):**

DERR1-10.2196/33894

## Introduction

### Background

According to the World Health Organization, the number of people aged 60 years or older will rise from 900 million in 2015 to 2 billion by 2050 [1]. Difficulties related to mobility frequently appear with aging. For example, a decline in physical abilities, such as balance [2], may increase risks when walking, such as falling on icy sidewalks or not having enough time to cross the road at traffic lights, or when using public transportation, such as when getting on and off the bus. A decline in cognitive abilities [3] and lack of digital literacy [4] can lead to difficulty planning outings and understanding public transportation options. Poor vision, slow reaction time, and reduced range of motion interfere with safe driving, an important symbol of autonomy in North America [2,5,6]. In addition, having few financial resources [7] and living in a rural area [8] make access to taxi and bus services more problematic. Many older adults have a small social network, which limits the number of people they can ask for help when they want to go out. These issues also increase the risk of being sedentary and isolated [9]. It is now well established that social participation is a significant determinant of older adults’ health and quality of life. Social participation may be defined as a person’s involvement in activities providing interactions with others [10] in community life and in important shared spaces; social participation evolves according to the available time and resources, with the societal context, and with what individuals want and what is meaningful to them [11]. Recent studies show that isolation is detrimental to older adults’ physical and mental health and leads to similar or even greater mortality risks than smoking and obesity [12]. Older adults’ ability to get to desired locations, such as the grocery store, hospital, or theater, is a vital aspect of their independence and an essential vector of their social participation [13].

Although options other than driving are available, such as taxis and buses, these means of transportation are not necessarily familiar to older adults, are sometimes not used by them, and are associated with various fears, such as getting lost, not being able to find a place to sit, or having unwanted social contacts [9]. Indeed, safety is one of the main concerns for older adults when using public transportation [14]. The recent COVID-19 pandemic revealed how the fear of public transportation could increase social isolation. Moreover, older adults often have different travel patterns from younger adults, because lifestyle transitions and age-related changes influence their needs, destinations, and travel time [15]. Shrestha and colleagues [14] reported that, ideally, transportation services for older adults should be accessible, affordable, comfortable, door-to-door, and available at a frequency that allows for spontaneous use and the ability to get to a wide range of destinations. It is therefore important to help older adults regain and sustain their postpandemic daily and community-based activities by improving the use of mobility options that are both flexible (usable where and when they want) and safe (reducing the risk of falls, incidents, and travel-related anxiety). For example, going to a doctor’s appointment might include being able to go downstairs, remain calm despite the stress of not arriving at the hospital on time, and find one’s way to the doctor’s office, which may require physical assistance, comfort, or help with navigating. Transportation for seniors should thus go beyond help leaving the house: it must ensure that they are independent and free to choose where, when, and how to get to desired locations while reducing the physical and psychological risks and discomfort associated with moving around.

### Starting Point

In June 2018, a community-based workshop was organized to identify and respond to the challenges of aging in place and mobility in Sherbrooke, Canada. More than 50 people, including 20 older adults, participated in this workshop, which was co-organized by members of LIPPA, the French abbreviation for the Laboratoire d’innovations par et pour les ainés (the laboratory of innovations by and for older adults). LIPPA’s mission is to create a more inclusive society by developing innovative and sustainable solutions by and for older adults [16].

The findings from a community-based workshop supported by LIPPA guided the development of this study. Two goals emerged from the workshop: promoting better intergenerational housing and fostering greater mobility for seniors. Discussions on the latter goal produced innovative ideas regarding how to better meet older adults’ transportation needs. Participants highlighted the difficulty older adults face in getting information about the different services available and knowing when and how to use them. They expressed the need to give older adults access to existing transportation services through a one-stop platform. Participants also stressed the need to speak with a real person; this aspect should be considered when designing the platform to ensure it is used by older adults who are not familiar with the technology. The gap in access to transportation services to support social and community-based activities, as well as the importance of building solutions based on existing services to avoid duplication, are consistent in reviews of the literature [13,17-20]. Thus, at the end of this workshop, researchers from LIPPA joined forces to address these needs.

### Objectives

The aim of this study is to develop Mobilainés (a portmanteau of the French words for “mobility” and “older adults”), a mobility as a service (MaaS) platform [21] that incorporates different modes and forms of transportation services, to help older adults move around where, when, and how they wish. This study uses a “Living Lab” approach [22]. Living Labs foster the creation of environments where stakeholders form public-private-people partnerships to develop and test new technologies in real life contexts. To promote older adults’ functional autonomy and ability to make decisions based on their own values and realities, Mobilainés requires knowledge development and the combination of diverse experiences, which naturally calls for a Living Lab approach. Compared to other transportation planning tools [23], Mobilainés will be unique in supporting older adults as they use various means of transportation adapted to their needs and preferences. More specifically, it considers personalized parameters that are often neglected in existing transportation planning tools [24] (eg, lifestyle habits and feeling safe when taking a trip), in addition to more general information easily accessible in current databases (eg, time and location). At present, using app-based travel information is considered the most realistic way to provide real-time information to address the needs of older adults. However, a large number of older adults [23] do not find transportation planning apps easy or intuitive to use. Mobilainés thus aims to include a technological interface, such as the web, with human support (eg, via telephone) to take into account the variability in digital literacy in an aging population. The functionality of our approach will also consider the aging process and region-specific issues in terms of human machine interactions, such as by using enhanced image contrast and voice commands and providing options such as calculating the time according to walking speed, giving the proximity of bus shelters and benches (for rest), and avoiding outdoor stairs. For example, Mobilainés could be designed to help an 81-year-old woman living in an urban area find a seat on the bus or avoid walking on icy sidewalks while planning a trip to the grocery store. Mobilainés does not seek to create a new mode of transportation; the goal is to merge existing transportation options in the same one-stop location to increase their usability and help older adults make decisions by providing useful information and recommendations based on available data, such as the roads most frequently cleared of snow in Sherbrooke. To support older adults in selecting and using the means of transportation that best fit their needs and preferences, this study had 3 objectives. Objective 1: define older adults’ mobility needs and preferences in order to conceptualize the one-stop platform. Objective 2: cocreate a prototype of the one-stop platform. Objective 3: test the prototype with users in a real-life context.

## Methods

### Design and Procedures

With LIPPA’s support and expertise, a Living Lab co-design approach is being used for this study. Five key elements usually define the Living Lab approach [25-27]: (1) the cocreation process, (2) intersectoral collaboration, (3) engagement of citizen-users throughout the process, (4) open-system development of innovative solutions, and (5) real-life context. Mobilainés incorporates these elements as it brings together intersectoral partners, including transportation providers and various community organizations, to rethink how to plan trips with older adults so that they feel more comfortable and safer when moving around. To support the future use of Mobilainés [28], it is crucial to secure older adults’ commitment at every stage of the process. Engagement, seen as a process that fosters the motivation to carry out a common project [29], can be increased by communication activities that encourage productive dialogue [30]. Participants in this productive dialogue are able to be objective about their own conceptualizations and make connections with conceptualizations that also benefit others and the project. For example, a dialogue becomes productive when individuals are able to make new distinctions using communication methods that foster this objectivity, such as workshops, presentations, and the use of lay language to convey information.

### Research Team

Our multidisciplinary team is composed of researchers with expertise in rehabilitation, communication, computer science, social work, and telehealth. Two master’s students and two PhD students in communication, gerontology, and computer science are also involved in specific aspects of the study, in accordance with their academic fields and degrees. The first authors and the project coordinator are currently meeting biweekly to discuss data collection and analysis and present results, as well as coordinate experts’ and partners’ contributions and timelines for everyone concerned.

### Steering Committee

A steering committee of 8 stakeholders from the public, scientific, private, and community sectors, as well as older citizens, has been tasked with monitoring the study’s progress, reviewing data collection methods and results, and deciding and prioritizing design aspects of Mobilainés through each study phase, ensuring its sustainability and respecting the citizen-centered Living Lab approach. Governance and ethical issues, such as intellectual property, are also being discussed. The committee meets quarterly, though the frequency varies depending on need as perceived by the members. As full partners in the process, older adult members help keep this population’s perspectives at the center of the creative process and ensure that their emotions, experiences, and limitations are considered in all decisions [31-33]. The steering committee contributes to responding to the needs and expectations of every partner around the table. Sessions are held in person or via videoconference and are audiotaped for data collection purposes. To maintain long-term engagement, visual tools are used at each meeting and constantly updated to provide information on the phase and timeline of each objective and a summary of data collection and results. Committee members are also invited to complete a survey at the end of each meeting to monitor their engagement level. The results of each phase are presented at yearly meetings to obtain specific feedback and clarify the members’ willingness to continue contributing to the project.

### Co-design Workshops

To support a co-design process throughout the study, intersectoral partners and older adults are invited to participate in in-person workshops every 2 or 3 months [34] using different cocreation methods and tools. We are using LIPPA’s well-established connections with local organizations to recruit older adults who present a wide variety of mobility profiles, experiences, and needs. At the beginning of each co-design workshop, a short video explaining the objectives of Mobilainés is presented to help participants understand the project as they “build the unknown” [35]. A special effort is made to clarify the roles, needs and contributions of all partners [36]. During the workshops, it is crucial to pay attention to the way older adults are involved in the process [37]. Online collaborative tools, such as Miro (Miro LLC), are used and shared on screen so that participants can see how they contribute to the workshop objectives. These co-design workshops are led by researchers who are familiar with the cocreation process and are audio- and videotaped for data collection purposes.

### Study Phases

Because the Living Lab approach is not linear, the innovation design has 3 phases, with each phase including several iterative subphases. This design incorporates the 3 main phases typically used in Living Labs: exploration, experimentation, and evaluation [38]. 

#### Phase 1 (Exploration): Defining Older Adults’ Mobility Needs and Preferences in Order to Conceptualize the One-Stop Platform

The aim of Phase 1 is to support the conceptualization of the one-stop platform by documenting the realities, needs, and expectations of older adults and transportation service providers and their satisfaction with existing mobility planning tools. This exploration phase includes 5 subphases. Phase 1.1 will identify facilitators and barriers to the mobility of older adults in Sherbrooke, and more specifically, documentation of their experience with planning trips and moving around. Phase 1.2 will determine the status of current transportation services as well as existing trip planning platforms and their functionality (including their content and interface). Phase 1.3 will define the criteria for the ideal Mobilainés platform [28]. Phase 1.4 will identify potential functionality to enable meeting the identified needs. Finally, phase 1.5 will bring together the most relevant partners in order to create the ideal platform.

In order to utilize the most up-to-date knowledge and experiences, the research team is gathering (1) evidence from recent scientific and gray literature by performing 2 scoping reviews [39] to identify (a) facilitators and barriers to older adults’ mobility and (b) existing tools for user interface and experience; (2) data from surveys completed by the steering committee and older citizens (n=8) to prioritize the platform criteria; and (3) results from 3 steering committee meetings as well as 5 in-person workshops that include transportation service providers, community-based and public health stakeholders (n=8), and older citizens (n=8); telephone interviews are also being conducted with very frail adults (n=8). Sustainable environment experts are also involved, to assess how environmental factors and social awareness can have a positive impact on the choice of transportation methods.

#### Phase 2 (Experimentation): Cocreating a Prototype One-Stop Platform

The aim of Phase 2, also ongoing, is to produce preliminary versions of the components of Mobilainés (eg, functionalities of the web application and telephone support). The necessary experts will be gathered and the objectives of 5 subphases will be established. Phase 2.1 will be the development of 4 profiles of typical older-adult end users of the platform to produce personas based on data gathered in Phase 1 [40]. Phase 2.2 will be recruitment, with the help of our partners, of 12 older adults embodying these personas (these 4 personas reflect older adults’ differing needs and preferences, such as the level of use and access to transportation planning tools when planning trips and the frequency and assistance required to go out). Phase 2.3 will be the production of usage scenarios and mockups (static designs of the web application) to present Mobilainés’s potential functionalities (content and interface) and the different ways older adult end users might utilize them (Miro, an online collaborative whiteboard, will be used to support the ideation process and create a first mockup, which will then be programmed in React, a JavaScript library [Meta Platforms, Inc]). Phase 2.4 will be testing of the scenarios and mockups with older adults (n=12), who will rate the features of Mobilainés in relation to their needs and preferences. Phase 2.5 will be refinement of the features of the one-stop transportation planning service, based on the input of the older adults. Decision-making tools, such as the “new, useful, feasible” (NUF) matrix [41], will help the steering committee choose between several mockup options according to their novelty, usefulness (or satisfaction [42]), and feasibility.

In this phase, different scenarios representing realistic uses of Mobilainés will be created to refine its functionality (eg, its ability to display interactive maps showing the most age-friendly routes, the ease of use of its interface, and its inclusion of fall prevention workshops), the accessibility of its interface (eg, telephone support and use of technology), and to provide information about how the preliminary prototype matches older adults’ needs.

#### Phase 3 (Evaluation): Pretesting the One-Stop Platform Prototype

Phase 3 will include 5 subphases. In phase 3.1, technology experts involved in the project will implement the technology platform functionality of Mobilainés, based on the mockups and scenarios developed in phase 2. Prototyping of the Mobilainés platform will rely on the use of technology for data gathering (eg, timetables, real-time location information, and user-generated content) [43]. The Mobilainés MaaS platform uses data acquired from various sources, including OpenStreetMap [44], the Google Maps platform [45], the Société de transport de Sherbrooke (the Sherbrooke transportation company) [46], and open-source data from the City of Sherbrooke [47]). These data will be processed and utilized to meet older adults’ mobility needs. The literature on journey planning algorithms will be reviewed, and algorithms will be tested to select the algorithm best adapted to older adults’ needs and preferences, as identified in Phase 1. Based on the available data and identified needs, the algorithm will find the most well-adapted and personalized itinerary to the given destination. Service agreements and protocols for human support (eg, telephone) will also be determined. In phase 3.2, older adults matching the personas identified in Phase 2 will test the prototype’s usability in specific scenarios. Usability tests of the web application will be performed iteratively in 2 sessions (testing initial and revised versions) with targeted end users (n=8). Participants will test 3 usage scenarios in which they have to navigate the web portal interface and obtain travel information for a specific destination while inputting specific preferences. Interactions with the web portal application will be captured with screen recording software. Facial expressions and verbal comments will be recorded using an external camera. Eye movements (saccades and fixations) will be recorded with an eye tracking system (Invisible; Pupil Labs). Task performance in the 3 case scenarios (including aspects such as critical errors and the task completion rate) will be analyzed [48]. A retrospective think-aloud session with gaze path simulation [49] combining the captured streams (screen, face, and gaze) will be used to debrief the participants and identify usability issues. Eye tracking metrics (such as fixation duration) will be extracted from eye movements using Blickshift analytics (Blickshift GmbH) and triangulated with the debrief results. At the end of the session, participants will also complete the System Usability Scale [50], a questionnaire frequently used to measure efficacy and satisfaction with usability when users perform specific tasks, as well as a questionnaire based on the Unified Theory of Acceptance and Use of Technology model, used to measure the perceived performance, expectancy, facilitating conditions, and social influence of a new technology [51]. Participants will then be invited to join a focus group to further document their satisfaction (quantified as like or dislike) with the navigation, information, and sensory design. Phase 3.3 will involve refinement of the prototype according to the results obtained in phase 3.2. Phase 3.4 will be testing of the Mobilainés prototype in real environments. Older participants (n=30) will be recruited through collaboration with local organizations and assigned different mobility profiles (see phase 2) to reflect a variety of needs, preferences, and characteristics, such as gender, income, rural or urban living area, and physical limitations. More specifically, we will perform pre-post measures after 6 and 12 weeks to identify changes resulting from use of the prototype in (1) older adults’ habits for trip frequency and destination, (2) mobility experience, such as moving around satisfactorily, getting to activities at the desired time, and feeling safe and comfortable when moving around, (3) participation in meaningful activities, and (4) empowerment (Table 1). We will use a combination of qualitative methods, such as semi-structured interviews and logbooks, and quantitative methods, such as actigraphy, geolocation, and questionnaires [52]. The participants will be asked to record their use of Mobilainés and the occurrence of adverse events, such as the death of a proxy or a major health change, in a logbook. To minimize seasonal effects on the measures, the participants will be divided into 3 groups of 10. Each group will be tested during fall, spring, winter, or summer. With the participants’ consent, individual geolocation data will be collected using GPS watches; participants may deactivate this data capture upon request. GPS coordinates will be used to document travel details, such as time outside the home, distance and time of travel, and mode of travel, and correlate this with use of the Mobilainés prototype, which will record inquiries made by each user. To prevent potential personal identification, individual geolocation data will not be linked to a geographic information system nor be presented or summarized at the individual level in any communications presenting the results [53].

The final phase, phase 3.5, will involve discussion of ways to support the adoption and implementation of Mobilainés. More specifically, to facilitate the transition toward scaling up the technologies emerging from Mobilainés, public and private sector partners will support the steering committee in establishing sustainability modalities. This final phase will refine the pretested prototype and the protocols for providing services to older adults. It may also uncover gaps in current transportation services that need to be filled to ensure a satisfactory mobility experience (eg, an intersection with traffic lights might have insufficient time for an older adult with a walking aid to cross the street).

**Table 1:** Prepost measurement variables and assessment methods.

Variables		Habits	Experience	Social participation	Empowerment
**Quantitative measures**					
	Technology-based capture: actigraphy and geolocation [52]	✓			
	French Canadian version of the Life-Space Assessment Questionnaire [54]	✓			
	Health Care Empowerment Questionnaire [55]				✓
**Qualitative assessment**					
	Semistructured interviews		✓	✓	
	Logbooks		✓		✓

### Data Analyses

Matrices will be used to perform mixed method analyses of the multiple types of data generated throughout the iterative processes of conceptualizing and creating the prototype (objectives 1 and 2) and by the pre-post qualitative and quantitative tests of the prototype (objective 3).

#### Qualitative Analysis

The workshop data will be produced, explored, and thematized using support interfaces, such as boards, post-its, drawings, and models. This production, exploration, and thematization process is quite widespread in co-design approaches since it allows everyone’s point of view to be considered, for improvements to be made collectively, and for meaning to be derived while avoiding desirability and cognitive convergence biases [36]. Data are reviewed to identify points of agreement and disagreement, which leads to a better understanding of how ideas change during group discussions [35]. This process allows the research team to (1) identify themes or comments that are not captured during the workshops; (2) confirm and consolidate existing themes; (3) explore the robustness of the themes; and (4) validate the conceptual tightness of the themes [36]. Themes emerging from the workshops will be compared with a systematic analysis of the data to ensure the reliability and validity of the results. Thematic analyses of the qualitative data streaming from the steering committee meetings and individual interviews will also be conducted during each study phase, as well as during the scoping review (phase 1.2).

#### Quantitative Analysis

Descriptive analyses will be performed to determine participant characteristics and context, as well as to examine the quantitative data streaming from surveys and questionnaires used in each study phase. In addition, statistical analyses (paired *t* tests) will be conducted to explore changes in participants’ behavior as a result of using the prototype (pre-post measures—phase 3.4). SPSS (version 12; IBM Corp) will be used.

### Data Triangulation

Data sources will be triangulated to address each subphase objective. For example, data from surveys, workshops, and literature reviews on age-friendly technologies and transportation planning apps will be combined to support the identification of the best functionalities to include [56,57] in Mobilainés (ie, this information will be used to compile “dos and don’ts”) (phase 1.4). Triangulation of the quantitative and qualitative data reduces the biases associated with each of these methods. For example, for the final tests (phase 3.4), participants may not be aware that they travel as often or for as long as they do or they may hesitate to report behavior they are ashamed of (such as walking slowly or avoiding busy routes), but this can be captured by actigraphy or geolocation. On the other hand, these methods may not capture significant problems encountered by older adults when moving around (eg, if they need assistance to fold a walker and put it in a taxi). Matrices in Excel (Microsoft Corp) will be used to merge qualitative and quantitative data [58]. A research assistant will conduct this analysis, and at least one other research team member will covalidate the qualitative data.

### Ethical Considerations

The Research Ethics Committee of the Centre intégré universitaire de santé et de services sociaux de l'Estrie—Centre hospitalier universitaire de Sherbrooke (Integrated health and social services center of the Eastern Townships and the University of Sherbrooke hospital) approved the study in September 2019 (approval number 2020-3389). Older adults and other members of the steering committee are considered official partners in the project rather than research participants. A LIPPA coordinator is available to support their participation in the steering committee and other cocreation activities. To promote equity between partners and recognize the specific contribution of older adults throughout the project, the same financial compensation (to cover meeting-related transportation expenses) will be offered to all steering committee members and others involved in the co-design sessions [59]. Since the cocreation process is dynamic and iterative, the research team may ask the Research Ethics Committee to approve modifications to the protocol in phases 2 and 3.

## Results

This ongoing study began in September 2019. The first 2 phases will be completed by September 2022. Due to the pandemic, steering committee meetings were conducted online from March 2020 to June 2021, but all workshops took place in person, complying with public health measures. The study is scheduled for completion by mid-2023. Years 1 and 2 were used to define the mobility needs and preferences of older adults in order to conceptualize the one-stop platform. Year 3 was used to cocreate the prototype one-stop platform. Year 4 will be used to test the prototype with users in a real-life context. In the spirit of open innovation, LIPPA will also share the results of each phase on its website [16]. Possible solutions that were not used will also be disseminated to allow other knowledge users to explore their possible implementation in other contexts.

## Discussion

### Expected Findings

The development of this one-stop platform transportation planning service will use a participatory and innovative methodology. Our Living Lab approach differs from traditional research on aging by facilitating intersectoral knowledge sharing and co-design of innovative solutions by and with older adults. As this approach improves adoption of the resulting innovation and its acceptability to users, Mobilainés is expected to produce practical scientific and socioeconomic impacts that benefit both individuals and society. More specifically, Mobilainés seeks to provide multimodal interfaces, such as web and telephone support, and functions, such as helping users avoid icy paths and sidewalks, that are based on quantitative and qualitative data from users with various profiles. These innovations will help to improve responses to a wide range of needs (based on disability level and technological knowledge), preferences (such as the desire to move around in familiar areas or take part in social interaction when taking a trip), and resources (such as income or learning ability) of a broad spectrum of older adults. According to Metz [60], available, effective, and affordable transportation facilities provide access to people and places necessary to maintain good quality of life. By promoting transportation services more adapted to the needs of older adults, Mobilainés will contribute to their quality of life by enabling active participation in the community, such as going to the shopping center, grocery store, or theater, and other activities significantly impacted by the pandemic. Mobilainés is also expected to help meet the needs of older adults for socialization while reducing the harmful effects of preventable incidents and the less visible consequences of sedentary lifestyles, isolation, and exclusion. For example, Mobilainés could help (1) reduce falls and car accidents by helping older adults avoid icy routes and sidewalks, (2) reduce inactivity by enabling older adults to walk outside more often, (3) reduce loneliness by helping older adults meet people, and (4) reduce the stigma affecting some modes of transportation [61]. In a postpandemic context, after lockdowns increased deconditioning and broke social ties, it is crucial to address these risks while considering health issues. Finally, over the long term, Mobilainés may act as a catalyst to change the built environment and society. For example, data collected from users may point to a lack of bus shelters in a particular area; knowing this should help to improve older adults’ mobility experience and support an informed decision on their transportation options. Although studies on older adults’ mobility in smart cities have increased in recent years in the United States, Europe [62,63], and Asia [7], it is still an emerging research area in Canada.

### Strengths and Limitations

As Mobilainés is being developed in a Quebec community with a wide variety of older adult demographics (eg, low vs high income and urban vs rural location [64]), our findings could be applicable to other communities. Although older adults face barriers that may differ from one place to another based on the steepness of the terrain, traffic density, and available transportation services, their mobility needs are similar, including going shopping, visiting relatives, going to leisure activities or places of work, and volunteering. By focusing on the needs expressed in co-design sessions by a broad spectrum of older adults (eg, by including those with high or low technological literacy) and reporting the progress of the study in real time on our website [16], Mobilainés has great potential for transferability, as both the methodology and the solutions we develop are potentially reproducible and exportable [65,66]. In addition, as one of the main challenges of research innovation is sustainability after the study is complete [65,66], our research team is seeking to increase the survivability of Mobilainés by addressing this important issue with members of the steering committee in the initial phases of the study.

Although a Living Lab approach is well suited to developing innovative solutions to complex social issues [67,68], including aging, it poses some challenges and creates some tensions [69], especially when it involves more vulnerable populations or marginalized situations [70]. For example, Living Lab studies that focus on older adults must take into account slower rates of innovation, communication and relationship issues, and the locations and types of co-design workshops [71,72]. Thus, in addition to the use of simple visual tools and videos developed by our research team [16], some co-design workshops will take place (when allowed by pandemic health measures) in the facilities of local organizations to foster partner engagement and participation by older adults [72]. In the same vein, our findings could extend to informing future co-design approaches and methods used to involve and engage older people in the process of designing technologies, such as employing videos to introduce people to different stages of the design and development process.

### Conclusion

With the proliferation of technological solutions brought about by the pandemic, there is a need to ensure that the platform will not be just another innovation that older adults will not use. To avoid this potential pitfall, Mobilainés will merge existing transportation options to support the use of services currently in place instead of adding new ones. Co-created by and for older adults, Mobilainés should also support their future use of the platform and potentially reduce the costs associated with multiple development cycles. Mobilainés differs from current platforms by helping older adults enhance their mobility experience, knowing that a positive experience will keep them motivated to go out again, especially in a postpandemic context, where feeling safe may be even more important than before. Mobilainés thus aims to become a crossroads where experience, knowledge, and innovation meet with the goal of fostering autonomy and freedom in older adults’ decision-making regarding transportation while reducing the physical and psychological risks of being harmed when moving around. We live in an era in which re-engagement in the community needs to be supported, and Mobilainés will contribute to a more inclusive society by improving older adults’ access to transportation, now and in the future, and by accommodating their current and anticipated needs.

## Abbreviations

LIPPA: Laboratoire d’innovations par et pour les aînés
MaaS: Mobility as a Service
NUF: New, useful, feasible
